# Quality of Service Routing in Manet Using a Hybrid Intelligent Algorithm Inspired by Cuckoo Search

**DOI:** 10.1155/2015/703480

**Published:** 2015-10-01

**Authors:** S. Rajalakshmi, R. Maguteeswaran

**Affiliations:** Department of Computer Science & Engineering, Jay Shriram Group of Institutions, Anna University, Chennai, Avinashipalayam, Tirupur, Tamil Nadu 638660, India

## Abstract

A hybrid computational intelligent algorithm is proposed by integrating the salient features of two different heuristic techniques to solve a multiconstrained Quality of Service Routing (QoSR) problem in Mobile Ad Hoc Networks (MANETs) is presented. The QoSR is always a tricky problem to determine an optimum route that satisfies variety of necessary constraints in a MANET. The problem is also declared as NP-hard due to the nature of constant topology variation of the MANETs. Thus a solution technique that embarks upon the challenges of the QoSR problem is needed to be underpinned. This paper proposes a hybrid algorithm by modifying the Cuckoo Search Algorithm (CSA) with the new position updating mechanism. This updating mechanism is derived from the differential evolution (DE) algorithm, where the candidates learn from diversified search regions. Thus the CSA will act as the main search procedure guided by the updating mechanism derived from DE, called tuned CSA (TCSA). Numerical simulations on MANETs are performed to demonstrate the effectiveness of the proposed TCSA method by determining an optimum route that satisfies various Quality of Service (QoS) constraints. The results are compared with some of the existing techniques in the literature; therefore the superiority of the proposed method is established.

## 1. Introduction

Mobile Ad Hoc Networks (MANETs) are an up-and-coming type of wireless communication networks, in which mobile nodes link on an ad hoc basis. MANETs are capable of changing their locations by themselves as well possessing the ability to configure on their own, facilitating peer-level communications among mobile nodes independent of fixed infrastructure. The key challenge in constructing a MANET is to equip each node to continuously retain the information required to appropriately route traffic. In particular MANETs research focuses on modeling different algorithms which possess high capability in selecting the routing path(s) and can effectively accommodate the desired QoS [1]. This infrastructure results in a highly dynamic topology resulting in a challengeable task for the Quality of Service Routing.

Similar to the other well established wireless networks such as WiFi, GSM, and CDMA, MANETs will not provide reliable services and QoS (Quality of Service). Basically, QoS considered is an imperative component for evaluating the performance of MANETs and henceforth the boundaries on bandwidth, delay, bandwidth delay product, jitter, and packet loss are found to largely influence the QoS factors. Thus QoS routing not only demand a path from a source to a destination but also demands a route that fulfils the end-to-end QoS constraints, prescribed as bandwidth or delay or loss. The prime objective of the route selection method is then to recognize a route that is most likely to fulfil the QoS requirements [2]. The primary objective of a QoS routing strategy is to maximize the utilizations of network resources by determining routes which are appropriate for different types of constraints imposed on MANETs by various applications.

The QoS routing protocol can also work in a stand-alone multihop mobile network for real-time applications [3, 4]. The QoS requirements largely depend on some of the key constraints, namely, link constraint, path constraints, and tree constraints. The main key constraint is the bandwidth; similarly, end-to-end delay is the path constraint and delay-jitter is the tree constraint [5]. Thus, satisfying all these interdependent constraints with conflicting objective makes the problem more complex and robust techniques are required to solve the considered QoS problem with the said constraints being met. When problem with complexities are to be considered, the obvious choice becomes heuristic techniques rather than the deterministic methods. Thus past researchers adopted several heuristic based techniques to solve the QoS routing problems [5, 6]. This is necessary for more improvement of various routing protocols for MANETs, and each proposed protocol claims that the strategy proposed provides an improvement over a number of different strategies considered in the literature [7].

In [8], an AMRoute is presented which deals with specific unicast routing protocol and is made feasible for other different unicast protocols. In another work [9], an on-demand QoS routing is proposed, wherein the link-state multipath QoS routing constructs a network topology employing a link bandwidth at the destination. In [10], a protocol called PUMA for ad hoc networks to establish and maintain a shared mesh for each multicast group is presented. A multiconstraint QoS routing using a new single mixed metrics is discussed [11]. Reference [12] presents an ABMRS in MANETs, which makes use of a set of static and mobile agents for QoS routing [13]. Another proposal called Optimized Link State Routing Protocol was introduced in [14].

In addition to the above exact methods, the multiconstraint QoS routing was also addressed by several heuristic methods, which are reviewed briefly. In [15], a novel QoS routing algorithm based on Simulated Annealing (SA) is presented. Reference [16] discusses a novel bee colony optimization algorithm called bees life algorithm (BLA) to solve the QoS multicast routing problem; the key point here is three constraints which are delay, allowed jitter, and requested bandwidth which are taken into consideration. Similarly, a Tabu search [17] based method considering two important QoS constraints, bandwidth and end-to-end delay, is included. Again in [18, 19] QoS routing was effectively and efficiently addressed using a globally optimizing ant algorithm. Similarly, a new creative algorithm to find the path of the MANET with the bandwidth-delay-constrained multicast routing problem is proposed based on a harmony search (HS) algorithm [20].

Although several heuristic approaches are applied to QoS routing in MANETs, they almost individually solve this problem. Most of the above algorithms suffer from premature convergence, which leads to the introduction of hybrid methods [21]. Here, a PSO-GA method is hybridized with the strengths of both the particle swarm optimization (PSO) method and genetic algorithm (GA) method which compromises the overlapping between natural selection and social behavior which realizes an effective and efficient search for the solution. Similarly, in [22], new fuzzy genetic algorithm for QoS multicast routing is also discussed.

**Figure 1:**
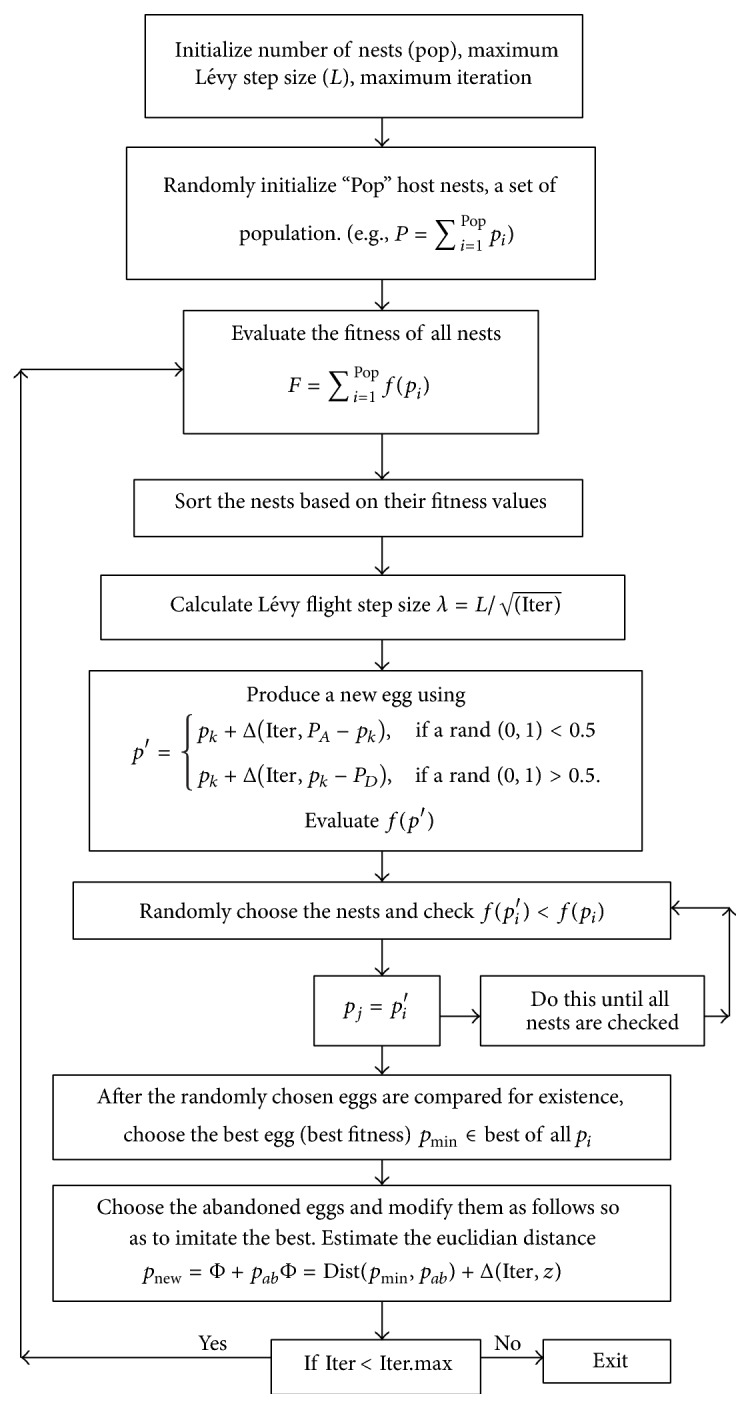
Flowchart of the proposed Tuned Cuckoo Search Algorithm.

Thus, in this paper, Tuned Cuckoo Search Algorithm (TCSA) method is proposed for the multiconstraint QoS routing problem (Figure 1). Cuckoo search (CS) [23] is one of the recent heuristic search algorithms inspired by the reproduction strategy. This low complexity of CS lends this new algorithm and its power to deal with complex and challenging problems [24, 25]. In this paper it is proposed again to improvise the performance of the CS method by introducing a new egg producing mechanism making the search of CS more exhaustive and leads to quick search of better solutions.

The rest of the paper is organized as follows. In Section 2, the problem statement of QoS routing problem in MANETS is introduced. The overview of CSA is briefly explained in Section 3 with description of the proposed tuning mechanism for the CSA.  Section 4 presents the simulation model and the numerical experiments with a standard QoS multicast routing problem on MANETs. This section also includes a detailed discussion about the proposed method in experimental perspective. The paper is concluded in Section 5.

## 2. Problem Formulation

The QoS routing problem is modeled as an optimization problem whose prime objective is to determine a multicast tree by taking into account the cost function which has to be minimized subject to some of the practical constraints. The present formulation considers four different constraints, namely, delay, packet loss, bandwidth, and jitter. The formulation is taken from [5] and for easy reference it is being presented here gain.

The objective function of the QoS routing is formulated as

(1) $$\begin{array}{c} \text{Minimize }C\left(H\left(x,S\right)\right)=C_{c}+\delta_{1}C_{b}+\delta_{2}C_{d}+\delta_{3}C_{dj}+\delta_{4}C_{pl}. \end{array}$$

The two most important reasons to combine both cost optimization and multiconstrained routing are the following:

1. Practically QoS routing problems are often formulated in terms of multiple constraints and the multicast structure is expected to identify a feasible path for each source and destination.
2. The significance of the network engineers and thus perfection of the users is to minimize the network resource utilization.

In summation, it can be represented as

(2) $$\begin{array}{c} C\left(H\left(x,S\right)\right)=\sum_{e\in H\left(x,S\right)}c\left(x\right)+\sum_{n\in H\left(x,S\right)}c\left(y\right). \end{array}$$

The objective is subject to be fulfilled satisfying the following constraints.

*The Delay Constraint (DELAY)*. It is the allowed delay limit along any branch of the network. A penalty is added if there is no link established between any two nodes. Consider

(3) $$\begin{array}{c} \text{DELAY}\left(R\left(x,y\right)\right)=\sum_{e\in R\left(x,y\right)}dl\left(x\right)+\sum_{n\in R\left(x,y\right)}dl\left(y\right). \end{array}$$

*The Bandwidth Constraint (BandWidth)*. It is the average bandwidth requirement of all the tree branches in the network. Consider

(4) $$\begin{array}{c} \text{BandWidth}\left(R\left(x,y\right)\right)=\min\left(bw\left(x\right)\right),\;x\in R\left(x,y\right). \end{array}$$

*The Delay-Jitter Constraint (DelayJitter)*. It is the maximum allowed delay variation in the entire network along any branch of the tree. Consider

(5) $$\begin{array}{c} \text{DelayJitter}\left(R\left(x,y\right)\right)=\sum_{e\in R\left(x,y\right)}dj\left(x\right)+\sum_{n\in R\left(x,y\right)}dj\left(y\right). \end{array}$$

*The Packet-Loss Rate*. Given the average packet loss along the tree branches normally will be 10%, violation will result in a penalty. Consider

(6) $$\begin{array}{c} \text{PackLoss}\left(R\left(x,y\right)\right)=1-\prod_{n\in R\left(x,y\right)}\left(1-pl\left(y\right)\right). \end{array}$$

Additionally, the components of the objective function are again formulated as follows:

(7) $$\begin{array}{c} C_{c}=\cos t\left(H\left(x,S\right)\right). \end{array}$$

Cost related to the bandwidth requirement is expressed as

(8) $$\begin{array}{c} C_{b}=\sum_{t\in S}\max\left\{Q_{B}-B\left(R\left(x,y\right)\right),0\right\}. \end{array}$$

Cost related to the delay requirement is expressed as

(9) $$\begin{array}{c} C_{d}=\sum_{t\in S}\max\left\{D\left(R\left(x,y\right)\right)-Q_{D},0\right\}. \end{array}$$

Cost related to the delay-jitter requirement is expressed as

(10) $$\begin{array}{c} C_{dj}=\sum_{t\in S}\max\left\{D_{J}\left(R\left(x,y\right)\right)-Q_{J},0\right\}. \end{array}$$

Cost related to the packet-loss rate is expressed as

(11) $$\begin{array}{c} C_{pl}=\sum_{t\in S}\max\left\{PL\left(R\left(x,y\right)\right)-Q_{PL},0\right\}. \end{array}$$

*Q*_D_ is delay constraint, *Q*_B_ is bandwidth constraint, *Q*_DJ_ is delay-jitter constraint, and *Q*_PL_ is packet-loss constraint. Additionally, *δ*_1_, *δ*_2_, *δ*_3_, and *δ*_4_ are the penalty constants of bandwidth, delay, delay-jitter, and packet-loss, respectively. In the next section, the proposed solution methodology using the modified Cuckoo search algorithm to solve the multicast QoS routing problem is explained in detail with numerical experiments.

## 3. Proposed Methodology

Solving a problem with complexities as shown above requires a solution technique that not only produces quality solution but also should be simple to implement. Due to increasing complexities of the problem in real time situations, this paper takes into account the possibilities of extending the proposed algorithm in future. Thus, this paper uses a Cuckoo search, one of the recent heuristic search algorithms, proven to be one of the best alternatives for the existing metaheuristic techniques. Before the proposed method is discussed, a brief overview of the Cuckoo search is presented.

### 3.1. Cuckoo Search (CS): An Overview

Cuckoo search is one of the recent heuristic search algorithms inspired by the reproduction strategy which differs from other host birds in the way that cuckoos choose nests of other birds to lay their eggs [26, 27]. Fortunately, the host bird easily distinguishes that the eggs are not its own and probably destroys the cuckoo egg. Thus, the cuckoo bird developed evolutionally produce eggs that look similar to the local host birds. Thus, three important points guide this as an optimization procedure, which are as follows:

- Initial Solution: the egg of cuckoo represents a set of solutions and its dimensions which are randomly placed at various nests.
- Next Generation: only a part of these eggs (the best eggs) with acceptable solutions will be allowed to move into the next generation.
- Acceptance Rule: if any of the eggs is identified as strange, then that particular (egg) solution will be removed and a new egg will replace this alien in a new nest.

The main steps of the CS are given below:

 Randomly Initialize “Pop” host nests, a set of population. (e.g., *P* = ∑_*i*=1_^Pop^ *p*_*i*_)
 Evaluate the fitness of all nests *F* = ∑_*i*=1_^Pop^ *f*(*p*_*i*_)
 Do Iterations < Max.Iterations
 Produce a cuckoo egg *p*_*i*_′ by applying a Lévy flight from a random nest
 Evaluate the fitness of *f*(*p*_*i*_′)
 Check for existence: choose again a random nest *j*
 if *f*(*p*_*i*_′) < *f*(*p*_*i*_) then
 
*p*_*j*_ = *p*_*i*_′
 end if
 Cull a fraction of the worst nests at the rate of *R*_*C*_
 Build new nests randomly at new locations via Lévy flights as replacements
 End While

A significant credit to this heuristics is its simplicity. This low complexity lends this new algorithm and its power to deal with complex and challenging problems. Perhaps when compared with other metaheuristic techniques such as particle swarm optimization and Ant colony algorithms, CS method has only a single parameter to be taken care of.

### 3.2. Tuning Procedure for the CSA

The original CSA is very simple to implement as it has less parameters to tune and hence there is a large probability for local convergence causing no quality results. Hence, this paper introduces a new tuning of the CSA by varying an operator that randomly changes the values at one or more search positions of the selected eggs. According to this new procedure, for each egg *P*_*i*_^*t*^ = {*p*_1_, *p*_2  _,…, *p*_*m*_} in the population of *t*th iteration, a new egg *P*_*i*_^*t*+1^ = {*p*_1_′, *p*_2_′,…, *p*_*m*_} is produced as below:

(12) $$\begin{array}{c} p_{k}^{\prime }=\left\{\begin{array}{cc} p_{k}+\Delta \left(\text{Iter},P_{A}-p_{k}\right), & \text{if a }\mathrm{rand}\left(0,1\right)<0.5\\ p_{k}+\Delta \left(\text{Iter},p_{k}-P_{D}\right), & \text{if a }\mathrm{rand}\left(0,1\right)>0.5, \end{array}\right. \end{array}$$

where *P*_*A*_ and *P*_*D*_ are the acceptance probability and declining probability rates of the variables *p*_*i*_. The quantity Δ(Iter, *y*) generates a real numbers in the range [0, *p*_*i*_] such that Δ(Iter, *p*_*i*_) approaches zero as number of* iterations* increases. Equation (12) makes the new eggs to search the space uniformly during the beginning of the run (when iteration number is small) and fine tune the search space as the iteration number progresses. This method of tuning increases the chances of producing new eggs close to its best survived eggs. The quantity Δ(Iter, *y*) is calculated using the following function:

(13) $$\begin{array}{c} \Delta \left(Iter,z\right)=z\left[1-{\xi^{\left(1-t/T\right)}}^{p}\right], \end{array}$$

where *ξ* is a random number between [0,1], *T* specifies the maximum number of iterations, and *p* is a system parameter that determines the degree of dependency on the generation number. This proposed new tuning operation is used to replace the regular updating of the eggs scheme used in the regular Cuckoo search algorithm and found this new mechanism will search complex space due to increasing constraints to the QoS problem and speed up the convergence rate.

Therefore, the proposed algorithm will generate the new nests and eggs of the run in such a way that the new egg should fulfil all the constraints. This is realized by taking into account the following criterion and is explained as follows:

- If the new egg structure is negative or zero, then replace the new egg's entire value by a newly generated random number which satisfies the constraint (problem dependent).
- If new egg structure is greater than one, then replace the new egg structure by one.

After the new nest is produced, it is evaluated by the fitness function.

## 4. Simulation Model

Numerical Simulations to demonstrate the applicability and performance of the proposed TCSA based QoSR algorithm are performed on a test system as briefed in Table 1. To evaluate the performance of the proposed method, an event-driven network simulator NS2 is used. To generalize the results and to ensure characteristics independence, this research uses a randomly generated network. In the simulation area we modeled a network of mobile nodes placed randomly within 1200 × 1200 square meters consisting of 50 to 100 randomly placed nodes. Nodes propagation range for each communication is 300 m and channel transmission capacity is set as 2 Mbps. Network partitions are neglected in the entire simulation. Each simulation time is restricted to 500 s. For the sake of comparison and to establish the superiority of the proposed method, this paper also compares the results of the proposed method with CS, PSO, and GA methods. 30 different trial runs with different initial values were performed and the averages of all the runs are summarized.

To evaluate the applicability of the proposed tuned Cuckoo search algorithm based routing protocol, the following performance metrics are used and the demonstration of the proposed algorithm is also compared with the other methods in this paper. The three methods use their own simulation parameters which are tabulated in Table 2.

*Packet Delivery Ratio*. The ratio of the number of data packets originally delivered to the receiver end to the number of data packets supposed to be received is evaluated as packet delivery ratio. This ratio provides information about how effectively the protocol is delivering packets to the receiver end. A large value of this ratio indicates the superiority of the proposed algorithm's performance and also confirms that more number of packets is delivered to the higher layers.

*Path Success Ratio*. The number of connectivity requests demanded by the receiver end to the original number of completed connections is termed as the path success ratio. This particular metric can only be evaluated when the proposed algorithm will be able to fulfil the delay and bandwidth constraints. The larger the ratio, the more effective the proposed algorithm for QoS routing.

*Average End-to-End Delay*. The delay in receiving packets indicates the performance of the network in transmitting packets from source end to destination end. The average end-to-end packet delay is calculated as the ratio of total end-to-end delays in the entire communication when compared with the number of packets successfully delivered to the receiver end nodes during the entire algorithmic run. A low value of this end-to-end delay means that MANET is less congested and subsequently witnesses the effectiveness of the proposed routing algorithm.

**Figure 2:**
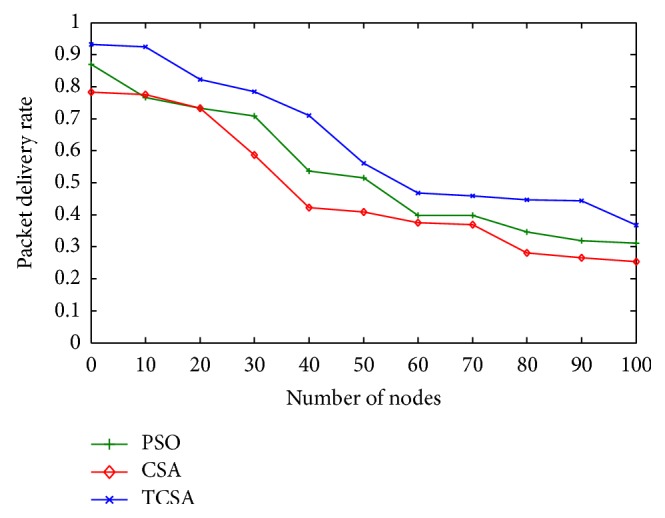
Plot between packet delivery ratio and network size.

**Figure 3:**
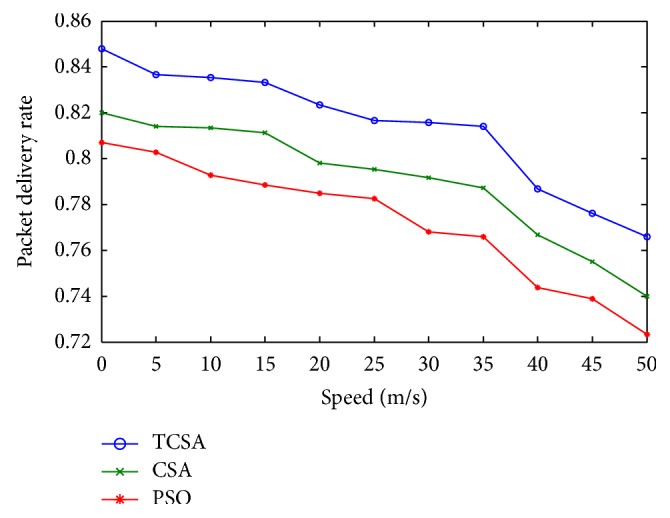
Plot of the real-time packet delivery ratio.

Subsequent to the numerical simulations performed using all the optimization procedures, the following observations are made with respect to the results comparing packet delivery ratio, path success ratio, and average end-to-end delay. Figures 2 and 3 show the performance analysis of the packet delivery ratio with respect to the network size for PSO, CSA, and the proposed TCSA algorithms in mobile ad hoc network. The result shows that values of the proposed TCSA algorithms are much better than PSO and CSA methods. This is because AODV needs to rediscover the route to retransmit data packets that are lost due to the node's mobility or unreal route paths during the communication. The advantage of FQRA resulted from choosing the right routing path or updating the unreal route paths just in time by the virtue of the suitable route lifetime estimation.

**Figure 4:**
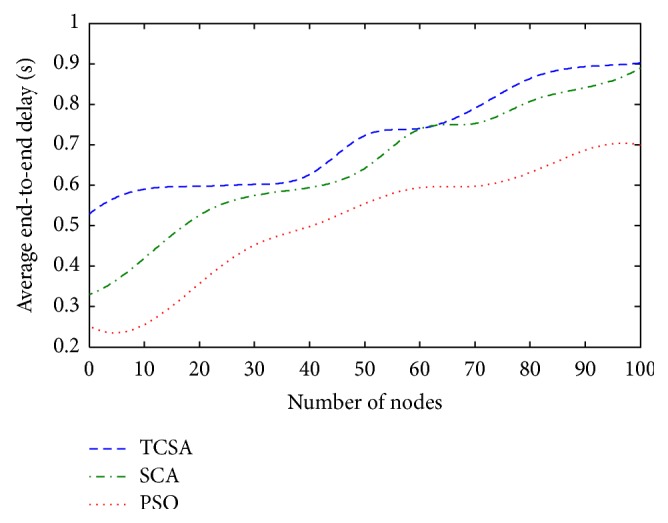
Plot between average end-to-end delay and network size.

Figure 4 shows the comparison plot of the three algorithms, PSO, CSA, and the proposed TCSA algorithms, with respect to the average end-to-end delay and the number of nodes. It shows that the end-to-end delay increases usually with the increasing node. From Figure 4, it can be seen that when the number of nodes increase, the TCSA algorithm average end-to-end delay is lower than that of PSO and CSA algorithm. Similarly, both PSO and CSA algorithms require more execution time and more control overhead than the TCSA method to recover unreal paths. When the number of nodes increases, the network receives more packets and thus end-to-end delay increases. The end-to-end delay is a significant factor as many practical applications demand small latency to deliver useful information within the prescribed period of time.

**Figure 5:**
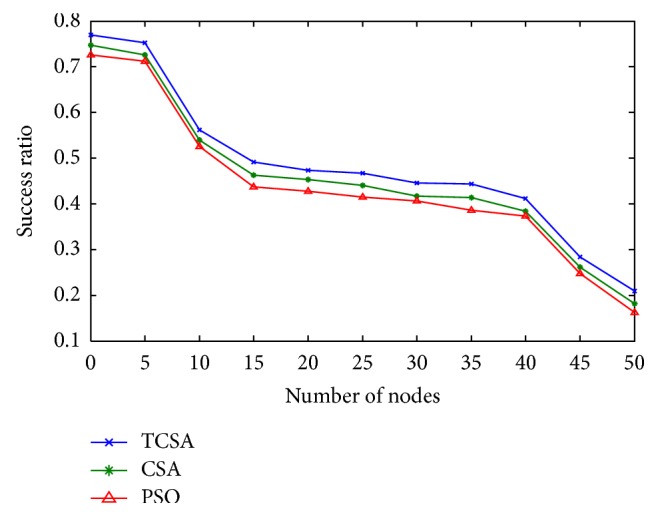
Plot between Success ratio of communication and network size.

**Figure 6:**
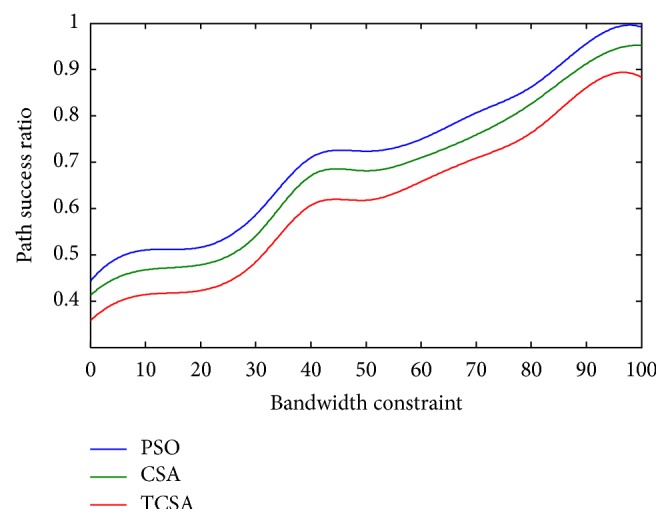
Plot between path success ratio and bandwidth.

Figures 5 and 6 summarize a comparison of the path success rate with respect to the number of nodes to find the path through PSO, CSA, and the proposed TCSA algorithms in mobile ad hoc network. The bandwidth constraints are ignored for the sake of comparison; it can be seen that the path success rate will be higher for CSA. Notably, the path success rate is further more than that of PSO, which confirms that it is more suitable for the routing selected based on data communication application and dynamic network topology.

**Figure 7:**
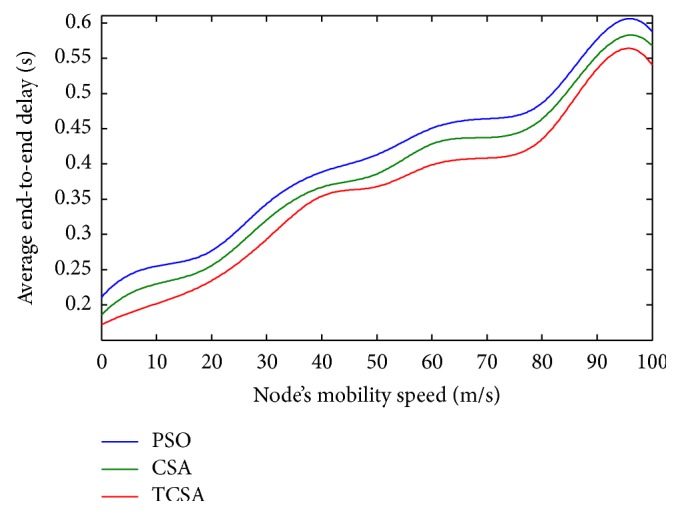
Plot between average end-to-end delay and node's mobility speed.

Finally, the average end-to-end delay plot with respect to the node mobility speed is shown in Figure 7. It can be witnessed that the end-to-end delay is better when delay jitter and packet loss rate are taken into consideration. It is ensured that the mobility is varied from 0–5 m/s; the proposed TCSA algorithm demonstrated that an end-to-end delay decreased by around 0.01 seconds to 0.03 seconds. It is also inferred that the end-to-end delay also increases normally with the increase in speed of nodes. It also shows that TCSA is far better than the other two routing methods, PSO and CSA. In addition, both CSA and PSO require more execution time than the proposed TCSA algorithm to discover new routes fulfilling all the QoS requirements.

## 5. Conclusion

In this paper, a QoS multicast routing method based on a Tuned Cuckoo Search Algorithm is presented. A hybrid computational intelligent algorithm is proposed by integrating the salient features of two different heuristic techniques to solve a multiconstrained Quality of Service Routing (QoSR) problem in Mobile Ad Hoc Networks (MANETs). The QoSR is always a tricky problem to determine an optimum route that satisfies variety of necessary constraints in a MANET. For the sake of showing the superiority of the proposed TCSA algorithm, additional simulations are carried out using the well-known PSO and CSA. Three most essential performance metrics, namely, packet delivery ratio, path success ratio, and average end-to-end delay are measured for the purpose of comparison with other methods. In all the challenging experiments, the proposed TCSA method proved to be more effective and efficient in producing superior QoS routing satisfying all the constraints put before it in the randomly generated mobile ad hoc network. Numerical experiments also demonstrate that the proposed hybrid TCSA algorithm can search better multicast trees with swift convergence with robustness compared with other algorithms. This proposed algorithm may also be extended for real-time networks with necessary modifications when and where required.

## Figures and Tables

**Table 1:** Test data for the randomly generated network.

Area of coverage by the nodes	1200 m × 1200 m
Nodes in the MANET	100
Communication range of the nodes	300 m
Initial energy in the network	8 J
Speed of movement of nodes	5 m/s
Pause time	0–430 s
Execution time	500 s
Number of trail runs	30
Packet size	512 bytes
Traffic density	12 pkts/s
Model of the mobility of nodes	Random waypoint

**Table 2:** Parameter settings for PSO and CSA optimization methods.

	PSO	CSA	TCSA
Particle size or nests	20	40	40
Maximum number of iterations	3000	3000	3000
Linear inertia weight	0.9 to 0.4	NA	NA
Levy's rate	NA	0.7	07
Termination condition	Fitness does not change for 20 iterations		
